# Treating oral mucositis with a supersaturated calcium phosphate rinse: comparison with control in patients undergoing allogeneic hematopoietic stem cell transplantation

**DOI:** 10.1007/s00520-012-1489-5

**Published:** 2012-06-27

**Authors:** Miroslaw Markiewicz, Monika Dzierzak-Mietla, Andrzej Frankiewicz, Patrycja Zielinska, Anna Koclega, Malgorzata Kruszelnicka, Slawomira Kyrcz-Krzemien

**Affiliations:** Department of Hematology and Bone Marrow Transplantation, Medical University of Silesia, Dabrowskiego 25, Katowice, 40-032 Poland

**Keywords:** Oral mucositis, Supersaturated calcium phosphate rinse, Hematopoietic stem cell transplantation, Graft-versus-host disease, Allogeneic transplantation

## Abstract

**Purpose:**

Of patients undergoing allogeneic hematopoietic stem cell transplantation (HSCT), 75 % or more experience oral mucositis, a painful acute complication that can delay discharge, interrupt treatment, and threaten life. To evaluate the efficacy of a supersaturated calcium phosphate rinse (SCPR), we compared it with customary care—topical mouth solutions—on measures of severity and consequent interventions and complications.

**Methods:**

In this randomized controlled trial, 40 patients undergoing allogeneic HSCT were randomized: 20 to SCPR four times daily and 20 to solutions made with salvia leaf extract, iodine-povidine, and fluconazole. Treatment extended from initiation of conditioning treatment until the granulocyte count was ≥0.2 g/L. Mucositis severity was measured daily by a hematologist according to a World Health Organization (WHO) scale and self-assessed by patients. Need for interventions [analgesics, total parenteral nutrition (TPN), and granulocyte colony-stimulating factor] and complications (acute graft-versus-host disease and infections) were also assessed.

**Results:**

In comparison with the control group, the SCPR group had significantly lower mean measures of WHO oral toxicity (0.9 vs. 1.8; *P* = 0.02), disease course (3.2 vs. 7.1 days; *P* = 0.02), and peak mouth pain (0.85 vs. 1.75; *P* = 0.005). Analgesic need was significantly shorter (1.1 vs. 3.4 days; *P* = 0.047) and the need for TPN significantly lower (0 vs. 6 patients; *P* = 0.02; 0 vs. 1.9 mean days; *P* = 0.009). Measures of complications were lower in the SCPR group, but not significantly so. Trial limitations include the impracticality of achieving double blinding with agents so different in appearance and in preadministration preparation.

**Conclusions:**

Compared with the control group, the SCPR group had significantly lower mean measures of oral toxicity, peak mouth pain, and disease course duration. These results warrant confirmation in controlled, multicenter, randomized trials.

## Introduction

Oral mucositis is the most common acute complication of hematopoietic stem cell transplantation (HSCT) conditioning regimens, and it has been reported to occur in 76–99 % of patients treated with high-dose chemotherapy and/or total body irradiation (TBI) before HSCT [1–3]. Mucositis is a result of both the direct toxic effects of chemotherapy and/or radiotherapy on mucosa and posttherapeutic neutropenia predisposing to viral, bacterial, and fungal infections. Mucositis, manifest in erythematous oral cavity ulcerations, can produce pain, dysphagia, xerostomia, changes in the voice, and life-threatening sepsis [4]. Affecting all functions of the mouth—drinking, eating, speaking—and dental and other mouth care, it affects not only nutrition and quality of life but also may necessitate total parenteral nutrition (TPN), demand morphine for pain relief, delay discharge, interrupt treatment, increase costs, and threaten life. Despite use of standard oral hygiene regimens, mucositis is one of the most common causes of severe pain in allogeneic HSCT recipients [5]. Drugs considered most harmful to oral mucosa include 5-fluorouracil, methotrexate, doxorubicin, etoposide, melphalan, cytarabine, and cyclophosphamide. TBI may also exert devastating effects on the mucosa [6].

Mucositis can be described by several scales, but the most common is the World Health Organization (WHO) five-stage scale: 0—no change; 1—soreness/erythema; 2—erythema, ulcers, patient can swallow solid food; 3—ulcers, patient requires liquid diet only; and 4—alimentation impossible [7].

Strategies for preventing mucositis include topical treatment with sodium bicarbonate in saline solution (NaHCO_3_/NaCl), chlorhexidine, hydrogen peroxide, and IB-367 (a naturally occurring antimicrobial agent derived from porcine neutrophil peptides) [8–12]. Positive effects have been obtained with glutamine, interleukin-11, keratinocyte growth factor (KGF), granulocyte or granulocyte macrophage colony-stimulating factor, and amifostine [13–19]. With colleagues, we have previously reported the beneficial influence of KGF (palifermin) on mucositis and acute graft-versus-host disease (GVHD) in a retrospective study using a historical control group [20].

In this study in patients undergoing allogeneic HSCT after high-dose chemotherapy and radiotherapy, we assessed the ability of the supersaturated calcium phosphate rinse (SCPR) Caphosol (EUSA Pharma, Langhorne, PA) to prevent mucositis, reduce its duration and severity, reduce the need for TPN and analgesics, and improve patient comfort.

## Patients and methods

This study was a prospective, randomized, nonblinded controlled trial with 40 consecutive patients undergoing allogeneic HSCT; half received treatment with the supersaturated rinse, and the remaining half received customary care with topical mouth solutions. Patients enrolled in this study underwent transplantation in the Department of Hematology and Bone Marrow Transplantation at the Medical University of Silesia in Katowice, Poland, in 2009. All patients provided written informed consent.

Medications were similar between the two groups. Patients were all on the same immunosuppressive therapy and were treated with the same antifungal, antibacterial, and antiviral agents during granulocytopenia. In the case of infection or suspicion of infection (neutropenic fever of unknown origin), therapy was chosen across both groups according to known or suspected microorganism without significant variation between them.

In this study, we tested the SCPR, a preparation consisting of two separately packaged aqueous solutions (a phosphate solution and calcium solution), as a preventive and treatment for mucositis. When both solutions are combined in equal volumes, a solution supersaturated with both calcium and phosphate ions is formed.

In the treatment group, patients rinsed their mouths four times daily with the SCPR; in the control group, patients received customary topical mouth care with extract of salvia leaves (twice daily), povidone-iodine mouth solution (1 % water solution of iodide with polyvinylopyrrolidone) once daily, and fluconazole mouth solution [fluconazole (50 mg), glycerin (50 mg), vitamin A (10 g), and vitamin E (10 g) with or without benzocaine (2.5 g)] twice daily. SCPR treatment was administered from the first day of conditioning until patients reached the absolute neutrophil count—≥0.2 g/L—a value that was considered an indication of the beginning of neutrophil recovery. Patients were stratified by age, preparative regimen (busulfan, treosulfan, or TBI), and type of transplant donor (related or unrelated) into two equal groups. Patients self-assessed the level of pain in the mouth and pharynx using a 0–10 visual analog scale (VAS) and measured swallowing problems using a 0–5 VAS [21]. The same experienced hematologist performed a physical examination of the oral cavity each day throughout the study, ranking cases according to the WHO scale for grading oral toxic effects of cancer treatment. Nonparametric Mann–Whitney *U* tests, Fisher exact two-tailed tests, and Yates chi-square tests were used for statistical analysis.

## Results

Eighty percent of the patients (32/40) had been diagnosed with leukemia (acute myeloblastic leukemia, 20; acute lymphoblastic leukemia, 10; and chronic myelogenous leukemia, 2; Table 1). The remainder had paroxysmal nocturnal hemoglobinuria (four), severe aplastic anemia (two), myelodysplastic syndrome (one), and osteomyelofibrosis (one). Almost half of the patients (18) had been on busulfan (16 mg/kg) and cyclophosphamide (120 mg/kg), 10 had undergone irradiation (12 Gy) and taken cyclophosphamide (120 mg/kg), and 10 had received treosulfan (42 g/m^2^) and fludarabine (150 mg/m^2^). One patient had received a higher dose of cyclophosphamide (200 mg/kg) and another one a higher dose of cyclophosphamide (160 mg/kg) with a lower dose of treosulfan (20 mg/m^2^).

**Table 1:** Patient characteristics

Characteristic	Supersaturated calcium phosphate rinse	Control^a^
Age (years)		
Mean (range)	38 (19–57)	36 (20–57)
Sex		
Men	13	11
Women	7	9
Regimen		
Busulfan (16 mg/kg) and cyclophosphamide (120 mg/kg)	9	9
Total body irradiation (12 Gy)/cyclophosphamide (120 mg/kg)	5	5
Treosulfan (42 g/m^2^) and fludarabine (150 mg/m^2^)	6	4
Treosulfan (20 g/m^2^)/cyclophosphamide (160 mg/kg)	0	1
Cyclophosphamide (200 mg/kg)	0	1
Source of transplant		
Sibling	5	4
Unrelated donor	15	16
Diagnosis		
Acute myeloblastic leukemia	8	12
Acute lymphoblastic leukemia	5	5
Chronic myelogenous leukemia	2	0
Paroxysmal nocturnal hemoglobinuria	3	1
Other (osteomyelfibrosis, myelodysplastic syndrome, severe aplastic anemia)	2	2

^a^Patients in the control group received topical mouth care with extract of salvia leaves and povidone-iodine and fluconazole mouth solutions

**Fig. 1:**
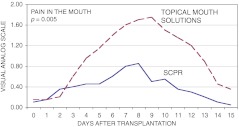
Mean ratings of pain in the mouth according to patients’ self-assessment using a visual analog scale. Values were significantly different (*P* = 0.005) (*SCPR* supersaturated calcium phosphate rinse)

Mucositis was evaluated by an experienced physician using the WHO scale (Table 2). The mean mucositis score for the SCPR group was 0.9, but it was twice that (1.8) for the control group (*P* = 0.02). The mean duration of mucositis was 3.2 days for the SCPR group, about half the 7.1 days for the control group (*P* = 0.02). Throughout the course of mucositis, average mouth pain intensity was significantly lower in the SCPR group than in the control group (Fig. 1); however, differences in average pain in the pharynx and with problems swallowing were not statistically significantly different. Measures of peak mean pain in the mouth, peak mean pain in the pharynx, and peak mean swallowing problems were all lower in the SCPR group (Table 2). Days to an absolute neutrophil count of >0.5 g/L and to a platelet level >20 g/L were not significantly different between groups.

**Table 2:** Measures of mucositis severity, interventions, and complications

Characteristics of mucositis and treatment	Supersaturated calcium phosphate rinse	Control^a^	*P*
	Severity		
Severity (WHO scale)	0.9 (0–4)	1.8 (0–4)	0.02
Duration (days)	3.2 (0–13)	7.1 (0–22)	0.02
Peak mean pain in mouth (0–10 VAS)	0.85	1.75	0.005
Peak mean pain in pharynx (0–10 VAS)	1.95	2.2	0.2
Peak mean swallowing problems (0–5 VAS)	1.1	1.6	0.3
Days to absolute neutrophil count >0.5 g/L	19 (12–29)	18.25 (12–31)	0.96
Days to platelets > 20 g/L	17.26 (9–31)	17.22 (8–34)	0.61
	Interventions and complications		
Analgesics	3	9	0.085
Duration analgesics used (days)	1.1 (0–13)	3.4 (0–18)	0.047
Total parenteral nutrition (TPN)	0	6	0.02
Duration TPN used (days)	0	1.9 (0–16)	0.009
Granulocyte colony-stimulating factor	0	4	0.106
Acute graft-versus-host disease (aGVHD)	7	9	0.747
Degree of aGVHD	0.5	0.9	0.3
Infectious complications	6	10	0.333

*WHO* World Health Organization, *VAS* visual analog scale

^a^Patients in the control group received topical mouth care with extract of salvia leaves and povidone-iodine and fluconazole mouth solutions

Interventions required by mucositis and related complications are reported in Table 2. In the SCPR group, no patient required TPN, but six in the control group required TPN. The average duration of TPN was 1.9 days in the control group versus 0 days in the SCPR group (*P* = 0.009). Analgesics administered for mucositis-related pain (ketoprofen, fentanyl, metamizole, buprenorphine, and acetaminophen) were required in three patients in the SCPR group but in nine patients in the control group. Average analgesic duration was 1.1 days (0–13 days) in the SCPR group, but 3.4 days (0–18) in the control group (*P* = 0.047).

Infectious complications following allogeneic HSCT were observed in five patients (25 %) in the SCPR group and in ten (50 %) of the control group. Of the five patients in the SCPR group, two had bacterial infections (one with bacteremia), one had a fungal infection, and two had cytomegalovirus (CMV) reactivations. Of the ten in the control group with infectious complications, six had bacterial infections (three with bacteremia) and four had CMV reactivations.

GVHD occurred in seven patients in the SCPR group (GVHD involvement: five, skin only; one, gut only; one, liver only) but in ten patients in control group (six, skin only; two, skin and gut; one, skin and liver; one, skin, gut, and liver). The mean overall degree of acute GVHD was 0.5 vs. 0.9, in favor of the SCPR group. None of these differences in GVHD between groups was statistically significant. The supersaturated rinse was well tolerated, no adverse events were observed, and no patient on it or in the control group withdrew early.

## Discussion

Mucosal damage is a devastating and debilitating complication of cytotoxic therapy that can have significant adverse clinical and economic consequences. In this trial, 40 patients undergoing conditioning for HSCT were randomized to two groups of 20 each: one group received treatment with SCPR and the other received routine care with extract of salvia leaves and antibacterial and antifungal solutions. Patients underwent SCPR treatments four times daily for an average of 14.6 (range, 8–21) days posttransplantation. Cases of mucositis in the treatment group were significantly less severe, had a significantly shorter duration, were associated with significantly less mouth pain, and required significantly less pain relief than those in the control group.

The distinguishing feature of SCPR in comparison with other mouth rinses is the high concentration of Ca^2+^ and PO_4_^3−^ ions. Theoretically, these highly concentrated ions exert their beneficial effect by diffusing into intercellular spaces in the epithelium of mucosa and permeating mucosal lesions. The Ca^2+^ ions play a crucial role in the inflammatory process, the blood-clotting cascade, fibrin production, and tissue repair. The PO_4_^3−^ ions also play an important biochemical role by facilitating intracellular signalling and regulating the voltage potential inside the cell, both important for repairing and protecting damaged mucosal surfaces [22]. The reduction of acidity in the oral cavity also may play a role in avoiding mucosa damage, creating a more favorable environment for opportunistic organisms, and diminishing mucositis symptoms.

The treatment used by the control group is a standard rinse that has been used within our center with good results for a long time. The components—salvia, povidone, and a fluconazole solution—are generally regarded as unproven. They were implemented against oral mucositis when it was formerly conceived as having a bacterial or fungal cause and are supported by research conducted with very small study groups, in trials of short duration, and often without true randomization or controls. They were certainly considered to be appropriate as controls and to be of no harm.

Salvia extract, which is thought to have antibacterial properties, was combined in one study with other agents in Chinese medicine and compared with Dobell’s solution (sodium borate, sodium bicarbonate, phenol, and glycerol) in treating 101 patients with advanced nasopharyngeal cancer undergoing chemoradiotherapy [23]. Neither statistically significantly outpaced the other in preset measures, including curative effects; however, no negative side effects were observed, and in a study of 24 patients with head and neck tumors who were receiving chemoradiotherapy, a mouthwash solution containing two species of salvia was associated with prevention of interruptions of therapy of 3 days or more [24].

Povidone-iodine, a disinfecting agent, is effective in decreasing bacterial oral cavity contamination [25, 26]. It was found in a randomized, prospective trial in 40 patients undergoing chemoradiotherapy for head and neck disease to reduce incidence, severity, and duration of oral mucositis significantly [27]. Two other trials compared povidone with water or saline and found no statistically significant differences for any of the outcomes [28, 29]. In 2004, povidone was listed by a consensus panel as having insufficient evidence to support a guideline [30], and in the panel’s update, it received no mention [31]. In 2011, a trial of 100 patients undergoing radiotherapy for head and neck cancer found benzydamine hydrochloride superior to chlorhexidine as well as povidone-iodine in delaying the progression of mucositis and reducing the pain, though differences were not statistically significant. In the second half of the same year, manufacturers initiated a recall of povidone-iodine at the request of the U.S. Food and Drug Administration. Products distributed nationwide were found to be contaminated [32].

Fluconazole, an antifungal agent, is employed to mitigate *Candida* oral cavity contamination [25]. It was found in a 2006 quality-of-life study of 63 patients with head and neck cancer undergoing radiotherapy to have significant beneficial impact as a prophylactic for oral mucositis severity, including preventing and reducing the presence of *Candida* infection [33]. Recent safety reports from the U.S. Food and Drug Administration have warned of birth defects caused by systemic use during the first trimester [34]. Reports were related to much higher doses (400–800 mg/day) than those topically applied in our study. In very rare cases, fluconazole has been associated with Stevens–Johnson syndrome [35, 36]. In these instances, doses were topical but two to four times that applied in this study.

Combined with fluconazole was glycerin, vitamin A, vitamin E, and sometimes benzocaine. Adverse effects associated with glycerin include inflammation associated with a glycerin-containing product [37] and reduced salivary amylase and pH levels when glycerin was combined with lemon [38]; however, the citric acid is thought to be responsible for the dramatic reductions. Another trial that included glycerin in combination with the herb payayor showed the combination was superior to benzydamine [39]. MuGard, a product recently approved in the USA and Europe for the palliation of oral mucositis pain, contains glycerin, and appeared to cause no known harm in trials; however, blinded, randomized trials have yet to be done in patients with head and neck cancer [40].

Both vitamin A and vitamin E have been reported as beneficial in oral mucositis. Mills et al. [41] demonstrated that 10 patients receiving vitamin A for chemotherapy- and radiation-induced oral mucositis developed less severe mucositis than ten who did not receive vitamin A over 10 to 12 patient-weeks, and researchers studying 80 pediatric patients in a 5-day study reported that 100 mg of vitamin E applied twice daily produced a significant benefit over supplementation [42]. Though not a study in oral mucositis, a 2010 single-blinded controlled surgical study in 428 pediatric patients found that vitamin E applied before and after surgery improved wound healing and cosmetic results [43]. Reports of topical vitamin E–induced contact dermatitis are rare, according to researchers who conducted a literature review and concluded it should not be removed from products used on the skin [44].

Benzocaine, which was employed with some patients in this study, is a widely used topical anesthetic. Benzocaine sprays used to numb the mouth for medical procedures have been identified by the U.S. Food and Drug Administration to be responsible for rare but serious adverse effects, including death [45]. Methemoglobinemia has resulted in some cases, and the U.S. Food and Drug Administration said that more than half of reports that included data on administration indicated overuse. It has not required safety warning labeling [45].

Apart from the contaminated products (which were not used in our trial), reported adverse events when povidone-iodine is allowed to pool and remain on exposed skin for long periods of time, overdosing, and other uncommon adverse events, we have no reason to believe these agents pose any danger to patients when used and monitored appropriately. The median duration of oral mucositis in the control group (7.1 days) compares favorably to data reported by others (7.2–8.0 days) [22, 46, 47], indicating if anything a competitive course duration.

The SCPR rinse is indicated as an adjunct to normal oral care in preventing and treating mucositis resulting from irradiation or high-dose chemotherapy, and it is also indicated for temporary or permanent dryness of the mouth and oropharynx (hyposalivation and xerostomia), no matter the cause [48]. Relief of dryness of the oral mucosa in these conditions is associated with amelioration of pain.

The trend toward lower incidence and severity of acute GVHD in the SCPR group may be related to decreased mucosal injury by chemotherapy or radiotherapy in this group of patients. An additional benefit in the SCPR group was the absence of the need for GCSF administration, although no significant differences in time to reconstitute granulocytes and platelets were observed between the groups.

Limitations of the study include the impracticality of maintaining a double-blind trial. Iodine solutions (red/orange color) contrast dramatically with the supersaturated calcium phosphate solution (colorless), making it difficult to disguise the differences. Furthermore, the phosphate solution has to be mixed and requires opening a clear ampule and a blue ampule immediately before administration.

The findings in this prospective randomized, controlled study confirm findings in a 1992 report of a double-blind prospective randomized controlled trial of 95 patients undergoing HSCT [22]. In that trial, SCPR produced statistically significantly lower measures of pain duration, disease course duration, use of analgesics (morphine), and duration of time to absolute neutrophil recovery than did a fluoride rinse, demonstrating that the SCPR regimen has a significant positive effect on oral mucositis associated with chemotherapy and radiotherapy. Under way currently is a randomized, placebo-controlled, double-blind multisite trial of SCPR by the Children’s Oncology Group (NCT01305200). The National Cancer Institute–funded study, which is expected to enroll 200, has as its primary outcome measure the duration of severe oral mucositis (WHO grade 3 and 4). One of several secondary measures will be the evaluation of a new pediatric oral mucositis scale, the Children’s International Mucositis Evaluation Scale. Findings are eagerly anticipated.

In the trial reported here, the SCPR mouth rinse was associated with decreased oral toxicity, including lower peak mouth pain and a shorter disease course, than were routine oral therapies. In consequence, in comparison with parallel values in the control group, the SCPR group had data indicating patient comfort was improved, the trend of acute GVHD hallmarks was lower, the requirement for TPN was diminished, and analgesic use was reduced. These results warrant confirmation in controlled, multicenter, randomized trials. Additional expanded investigations of its use in larger trial populations and in other settings in which mucositis occurs should be considered.
